# The Australian Paradox

**DOI:** 10.3390/nu4040258

**Published:** 2012-04-10

**Authors:** Peter Howe

**Affiliations:** *Editor-in-Chief of Nutrients*, Nutritional Physiology Research Centre, Sansom Institute for Health Research, School of Health Sciences, University of South Australia, Adelaide, South Australia 5001, Australia; Email: peter.howe@unisa.edu.au; Tel.: +61-8-8302-1200; Fax: +61-8-8302-2178

*Nutrients* recently became the target of an unprecedented internet campaign by an individual who disagrees with the content and conclusions of a paper published in the journal last year, viz. “The Australian Paradox: A Substantial Decline in Sugars Intake over the Same Timeframe that Overweight and Obesity Have Increased” by Alan W. Barclay and Jennie Brand-Miller, *Nutrients* **2011**, *3*, 491–504. Regrettably, his criticism has extended to the journal and its peer review processes for permitting publication of the article.

As you may know, *Nutrients* is one of an extensive series of on-line open access journals published by MDPI, who abide by internationally accepted standards of anonymous peer-review publication. Moreover, as one of the first MDPI journals addressing a field of biomedical/clinical sciences, our editorial team has endeavoured to adopt all appropriate conventions regarding ethics approvals, clinical trial registrations and declarations of perceived conflicts of interest. I have been grateful for the efforts made by members of the MDPI editorial team, our editorial board, our reviewers and our contributors for helping to ensure that the desired standards of publication are attained. I believe these standards were applied to the review of the paper in question and, despite inferences to the contrary, neither author had a role in the editorial process.

*Nutrients* does not have a policy of inviting correspondence to the Editor, nor has the journal received any formal correspondence regarding this manuscript. However, in view of the widely circulated criticism of the paper by Barclay and Brand-Miller, I believe that it is in the interest of the journal as well as the authors to afford them an opportunity to address these criticisms and provide further clarification of their research. This correspondence now appears on the Nutrients website at http://www.mdpi.com/2072-6643/3/4/491/.

I will leave our readers to judge for themselves.

